# Machine learning algorithms to predict intraoperative hemorrhage in surgical patients: a modeling study of real-world data in Shanghai, China

**DOI:** 10.1186/s12911-023-02253-w

**Published:** 2023-08-10

**Authors:** Ying Shi, Guangming Zhang, Chiye Ma, Jiading Xu, Kejia Xu, Wenyi Zhang, Jianren Wu, Liling Xu

**Affiliations:** 1 Hongqiao International Institute of Medicine, Tongren Hospital, Shanghai Jiao Tong University School of Medicine, 1111 XianXia Road, Shanghai, 200336 China; 2 Department of Anesthesiology, Tongren Hospital, Shanghai Jiao Tong University School of Medicine, 1111 XianXia Road, Shanghai, 200336 China; 3 Shanghai Institute of Computing Technology, 546 YuYuan Road, Shanghai, 200040 China

**Keywords:** Intraoperative hemorrhage, Machine learning, Gradient boosting decision Tree, LGBoost

## Abstract

**Background:**

Prediction tools for various intraoperative bleeding events remain scarce. We aim to develop machine learning-based models and identify the most important predictors by real-world data from electronic medical records (EMRs).

**Methods:**

An established database of surgical inpatients in Shanghai was utilized for analysis. A total of 51,173 inpatients were assessed for eligibility. 48,543 inpatients were obtained in the dataset and patients were divided into haemorrhage (N = 9728) and without-haemorrhage (N = 38,815) groups according to their bleeding during the procedure. Candidate predictors were selected from 27 variables, including sex (N = 48,543), age (N = 48,543), BMI (N = 48,543), renal disease (N = 26), heart disease (N = 1309), hypertension (N = 9579), diabetes (N = 4165), coagulopathy (N = 47), and other features. The models were constructed by 7 machine learning algorithms, i.e., light gradient boosting (LGB), extreme gradient boosting (XGB), cathepsin B (CatB), Ada-boosting of decision tree (AdaB), logistic regression (LR), long short-term memory (LSTM), and multilayer perception (MLP). An area under the receiver operating characteristic curve (AUC) was used to evaluate the model performance.

**Results:**

The mean age of the inpatients was 53 ± 17 years, and 57.5% were male. LGB showed the best predictive performance for intraoperative bleeding combining multiple indicators (AUC = 0.933, sensitivity = 0.87, specificity = 0.85, accuracy = 0.87) compared with XGB, CatB, AdaB, LR, MLP and LSTM. The three most important predictors identified by LGB were operative time, D-dimer (DD), and age.

**Conclusions:**

We proposed LGB as the best Gradient Boosting Decision Tree (GBDT) algorithm for the evaluation of intraoperative bleeding. It is considered a simple and useful tool for predicting intraoperative bleeding in clinical settings. Operative time, DD, and age should receive attention.

**Supplementary Information:**

The online version contains supplementary material available at 10.1186/s12911-023-02253-w.

## Introduction

Hemorrhage represents a major, life-threatening intraoperative complication that concerns any surgeon [1, 2]. Intraoperative bleeding worsens the quality of the surgical field, extends the time of the procedure, and increases the risk of complications [3]. Major bleeding requiring transfusion is associated with increased mortality and cardiovascular complications including myocardial injury and infarction, stroke, and acute kidney injury [4, 5]. The incidence of intraoperative bleeding varies greatly; that of endoscopic procedures can range from 2.9–45.1% [6], and that of presacral hemorrhage of rectal cancer can range from 4.6 to 9.4% [7].

In previous research, various risk factors have been associated with intraoperative bleeding. For example, increased operative time and abnormal erythrocyte size have been linked to intraoperative blood loss in orthopaedic surgery [8]. Low serum albumin levels and intraoperative platelet counts have been identified as potential risk factors for perioperative bleeding in gastrointestinal surgery [9], and a higher Body Mass Index (BMI) has been associated with increased bleeding in cardiac patients [10]. However, these studies are often limited by their focus on specific surgeries or patient groups, and they tend to ignore factors related to the operator. To address these limitations, we used real-world data from all inpatients to develop our perioperative bleeding models, with the aim of identifying potential risk factors that are more broadly applicable.

Previous studies usually developed traditional prediction models with intraoperative bleeding identified by univariate and multivariate logistic regression analyses [11, 12] without considering the nonlinear relationship or the multicollinearity of variables. Compared with traditional statistics, machine learning algorithms have fewer restrictions on data and can build complex data modeling [13]. Machine learning algorithms have also demonstrated promising performance for imbalanced real-world data (RWD). These data do not need to be specifically collected by health care providers [14], and the increasing availability has made it a crucial exploration in the generation of clinical insights [14]. Several studies have explored the potential benefits of combining traditional statistical methods with machine learning algorithms. For example, some researchers have used hybrid machine learning systems (HMLS) that combine dimensionality reduction algorithms and survival prediction algorithms to improve the accuracy of survival predictions [15]. Other studies have used a combination of logistic regression analysis and machine learning algorithms, such as extreme gradient boosting (XGB), and artificial neural networks 3 (ANN3), to construct prediction models for intraoperative blood transfusion [13]. Similarly, Eskandar Taghizadeh et al. identified the most optimal HMLS for diagnosing breast cancer, which included feature selection algorithms, a feature extraction algorithm, and classifiers [16]. These approaches demonstrate the potential for integrating traditional methods with machine learning algorithms to improve the accuracy and effectiveness of prediction models. However, HMLS still has the shortcomings of traditional statistical models. Therefore, this study attempts to use machine learning models completely to simulate real-world intraoperative bleeding situations and solve common overfitting and data imbalance problems in previous models. They included light gradient boosting (LGB), extreme gradient boosting (XGB), cathepsin B (CatB), Ada-boosting of decision tree (AdaB), logistic regression (LR), long short-term memory (LSTM), and multilayer perception (MLP). A table with an overview of the forecast models has summaried below (Table 1).

**Table 1:** An overview of the forecast models

Model	Describe	Strengths
AdaB	an ensemble learning algorithm by iteration until a stop condition is reached or the error rate becomes sufficiently small [27].	the ability to handle complex datasets and feature interactions
LGB	based on gradient boosting decision trees	optimize training speed and memory usage
XGB	a boosting integrated machine learning algorithm based on the CART regression tree.	integrates regularization techniques and feature selection methods, demonstrating strong generalization ability and predictive performance [17].
CatB	a gradient boosting machine learning algorithm	high performance in categorical features
LR	a supervised learning method and a member of the general linear model family [16]	simple
LSTM	a supervised recurrent neural network	capture time correlation more effectively [16].
MLP	one of the simplest artificial neural networks (ANNs) for data classification tasks [17] [17].	suitable for solving classification and regression problems

The assessment of bleeding risk during the perioperative period has long been a focus of clinical and research attention. While many specialties have developed bleeding risk assessment scales, the standards are not unified. For example, the intervals between hemoglobin concentration groups before surgery differ [18], and these scales cannot be updated in a timely manner with the development of surgical techniques. Pre-operative medication can reduce the likelihood of bleeding. The combination of vasopressin and nitroglycerine can substantially reduce hepatsplanchnic blood flow, but this treatment is only suitable for specific liver surgeries [19]. Tranexamic acid is another drug that has been shown to potentially reduce surgical bleeding, but it has also been associated with an increased risk of venous thromboembolism [5]. To gain a better understanding of the risk factors and underlying mechanisms of intraoperative bleeding, we took an observational approach. We placed inpatients in a natural state without any intervention and observed the potential association of certain factors with bleeding. This observational study design allowed us to capture a wide range of patient and clinical factors that may be associated with bleeding and identify potential confounding variables that may need to be accounted for in subsequent analyses.

## Methods

### Subjects

This study was conducted on surgical inpatients who were admitted to a single tertiary hospital (Tongren Hospital affiliated with Shanghai Jiao Tong University School of Medicine) from 1 to 2017 to 31 December 2021.

**Fig. 1:**
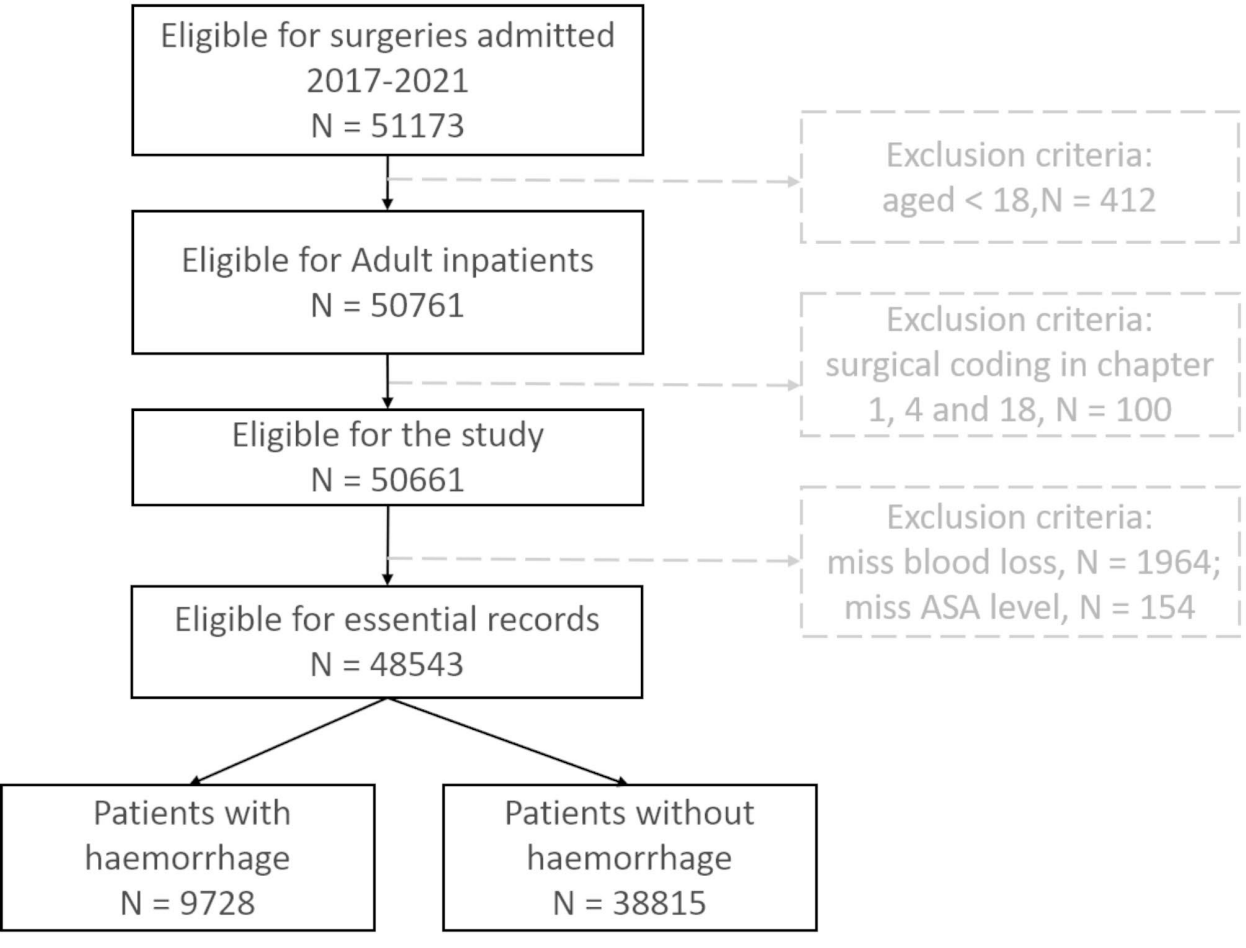
Flow chart of the study strategy. N, number of patients

Participants met the inclusion criteria: age ≥ 18 years. The exclusion criteria were as follows: (1) surgical coding in Chap. 1 (operations and interventions), Chap. 4 (operations for eyes) and Chap. 18 (various diagnostic and therapeutic procedures) of the International Classification of Diseases Clinical Modification of 9th edition; (2) uncountable bleeding volumes; and (3) loss of baseline information. The inclusion and exclusion criteria for our study are outlined in Fig. 1. Out of a total of 51,173 inpatients, 412 were younger than 18 years and 100 were excluded due to undergoing simple surgeries or diagnostic procedures with minimal bleeding. Additionally, 1964 inpatients were missing blood loss data, and 154 were missing ASA level data. After accounting for these exclusions, we ended up with a dataset of 48,543 inpatients. These patients were divided into two groups: those who experienced bleeding during the procedure (N = 9728) and those who did not (N = 38,815).

### Data collection

A data analysis and statistical plan was written and filed with the Changning District Committee of Science and Technology before data were accessed. The outcome of the study was defined as intraoperative bleeding when the bleeding volume was over 200 ml or with a drop in hemoglobin ≥ 3 g/dL or hematocrit ≥ 10% (accounting for transfusions) [20]. The bleeding volume was obtained from the anesthesia record sheet and it is measured by the anaesthetist based on intraoperative bleeding by negative pressure suction and gauze weighing as an indirect estimate method. If a patient had two or more surgeries in one day, only the heaviest volume loss was considered.

The patients’ demographic characteristics and clinical and laboratory test findings were extracted from the medical center’s electronic medical records (EMRs). The personal information of the surgeons was obtained from the human resource system. These data were cleaned and checked manually. There were still some null values in the BMI, and K-Nearest Neighbor (KNN) [21] was selected to fill in the missing values. It identified neighboring points by a distance measure and can use the full value of neighboring observations to estimate missing values.

### Feature selection and data preprocessing

To select the variables for our study, we utilized a rigorous methodology that included several steps. Firstly, we conducted a systematic review of relevant studies to identify potential variables. Secondly, we consulted with experts in the field to ensure that our list of variables was comprehensive. Thirdly, we conducted initial univariate analyses to examine the association between each variable and the bleeding outcome. Finally, we used a combination of statistical significance and clinical relevance to select the final set of variables. The EMRs dataset included 27 variables: three clinical variables (age, sex, BMI), five underlying illnesses (kidney/heart/hypertension/diabetes/coagulopathy), six surgical variables (surgery coding, surgical level, emergency/elective procedures, anesthesia method, ASA, operative time), four surgeon variables (occupational title, departments, length of employment, academic degrees), and nine biochemical criteria (pulse, systolic blood pressure, blood glucose, D-dimers, hemoglobin, hematocrit, thrombin time, prothrombin time, partial thromboplastin time). In the course of patient admission, well-established features were chosen as input features for the model (Appendix Table 1). The anesthesia modality is organized in the form of appendix Table 2 [22, 23].

### Model development

Seven machine learning models that use different classifiers were developed to predict the occurrence of the outcome in the Introduction section. Boosting refers to the use of a series of linear combinations of models to complete model tasks. It includes AdaB [24] and gradient boosting [25]. In gradient boosting, there is a technique called GBDT whose base learner is CART (Classification and Regression Trees). LGB, XGB, and CatB are all GBDT algorithms. LSTM is a supervised recurrent neural network that can capture time correlation more effectively [26]. LR is a member of the general linear model family [26]. MLP is one of the simplest artificial neural networks (ANNs), which consists of three layers—an input layer, an output layer, and a hidden layer [27].

In our study, we employed the L1 regularization technique as the feature selection algorithm [28]. This technique encourages sparsity in the model by adding the absolute values of the coefficients as a penalty to the loss function. By doing so, the model is encouraged to select a subset of important features, which effectively reduces the dimensionality of the feature space. This approach allowed us to identify the most important features for our analysis and improve the performance of our prediction models.

**Fig. 2:**
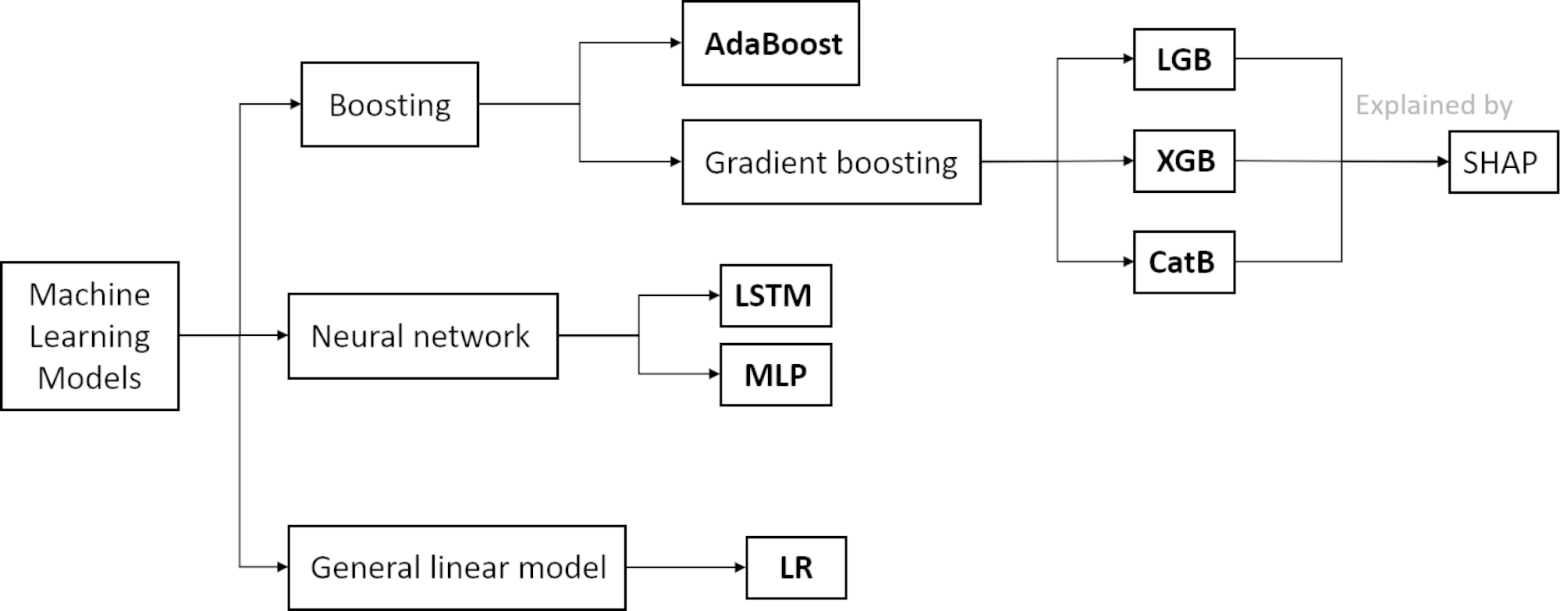
The relationships of applied maching learning models

We utilized the SHapley Additive exPlanation (SHAP) technique [29] to interpret predictions from tree ensemble methods, such as gradient boosting machines [30]. This technique allowed us to gain insights into how each feature contributed to the overall prediction and understand the relationship between features and outcomes. In Fig. 2, we demonstrated the relationship between SHAP and tree ensemble methods. By using this approach, we were able to gain a better understanding of the factors that contribute to perioperative bleeding and improve the interpretability of our prediction models.

All data were stored in a database (SQLite v3.16.0, http://sqlite.org/). Further analysis was performed using Python v3.8.3 with the lightgbm v3.3.2, xgboost v1.6.1, catboost v1.1, keras v2.10.0, scikit-learn v1.1.3, imbalanced-learn v0.8.1, and shap v0.41.0 packages (all available on https://cran.r-project.org/).

The dataset was randomly split into two dataset (2:1) cohorts, which were used to train machine learning models and tune their parameters, and a test cohort for model validation. The ratio of positive and negative samples was guaranteed to be the same in the training and test sets.

We found that the proportion of positive samples in the total dataset was approximately 1/4 and tried to use adaptive comprehensive oversampling Adaptive synthetic sampling (ADASYN) [31] to enhance the training data. The learning of the data distribution was improved in two ways by generating synthetic samples, reducing the bias caused by class imbalance, and adaptively moving the classification decision boundary towards instances where positive samples bleed.

To mitigate the risk of overfitting in our model, we implemented several best practices. Firstly, we carefully tuned the hyperparameters of the LGB algorithm, such as learning rate, maximum depth, and minimum data in leaf, using cross-validation and grid search. Secondly, we employed early stopping techniques during the model training process. Finally, we performed thorough model evaluation using various metrics, including precision, recall, and F1-score, to assess the model’s generalization performance on independent test data. By adopting these practices, we aimed to ensure that our model can effectively generalize to new data and produce reliable predictions.

Trials of seven machine learning classifiers were employed to generate models for the prediction of the study outcome. Model performance was assessed according to the area under the receiver operating characteristic curve (AUC), and the best-performing model was selected by AUC, sensitivity, specificity, and accuracy. The AUC reflects the discriminative power of these models, sensitivity represents the true positive recognition of patients with hemorrhage, specificity represents the true negative recognition without hemorrhage, and accuracy reflects the true recognition rate with both positive and negative results. Additionally, we used the AUC as our performance metric instead of accuracy, as accuracy can be misleading in the presence of class imbalance. The variable importance of each predictor for the optimal model was presented to rank their relative influence on a hemorrhage.

In the training process, it is more meaningful to identify high-risk patients of intraoperative bleeding than low-risk populations. We continuously adjust the recall rate and precision rate by reducing confidence and other operations. A random grid search was used to adaptively adjust the hyperparameters. The eigenvalue image degree of the results was also analyzed and the performance of the results was degraded.

### Statistical analysis

We reported categorical variables as counts (%) and continuous variables as means ± standard deviation (SD) or interquartile ranges (IDRs). The normal distribution was verified by the Kolmogorov-Smirnoff test.

Two-tailed t-tests were applied to compare baseline characteristics between continuous variables, the Mann-Whitney U test was used for nonparametric variables, and the χ² test was used for categorical variables. A two-sided *p* < 0·05 was considered statistically significant. All analyses were performed with SPSS version 24.0 (IBM Corp, Armonk, New York, USA).

### Ethical considerations

All participants were informed of the objectives, contents, potential risk and benefits of this survey prior to the data collection. Written informed consents were obtained from participants prior to study procedures. Study participants were assigned a unique identifier number to collect data confidentially. The present analysis was approved by the Ethics Committee of Tongren Hospital (2022-084-01).

## Results

The intraoperative bleeding data set of this paper was obtained, including 9728 positive samples and 38,815 negative samples (Fig. 1). The presence of hemorrhage occurred in 6518 (20.04%) patients in the training dataset and 3210 (20.04%) in the test dataset. The clinical and therapeutic characteristics of the study population are shown in Table 2. The mean age of all subjects was 52.61 ± 17.33 years. Males accounted for 57.5%, and females accounted for 42.5%. There were no predictors with a significant difference between the training set and the test set.

**Fig. 3:**
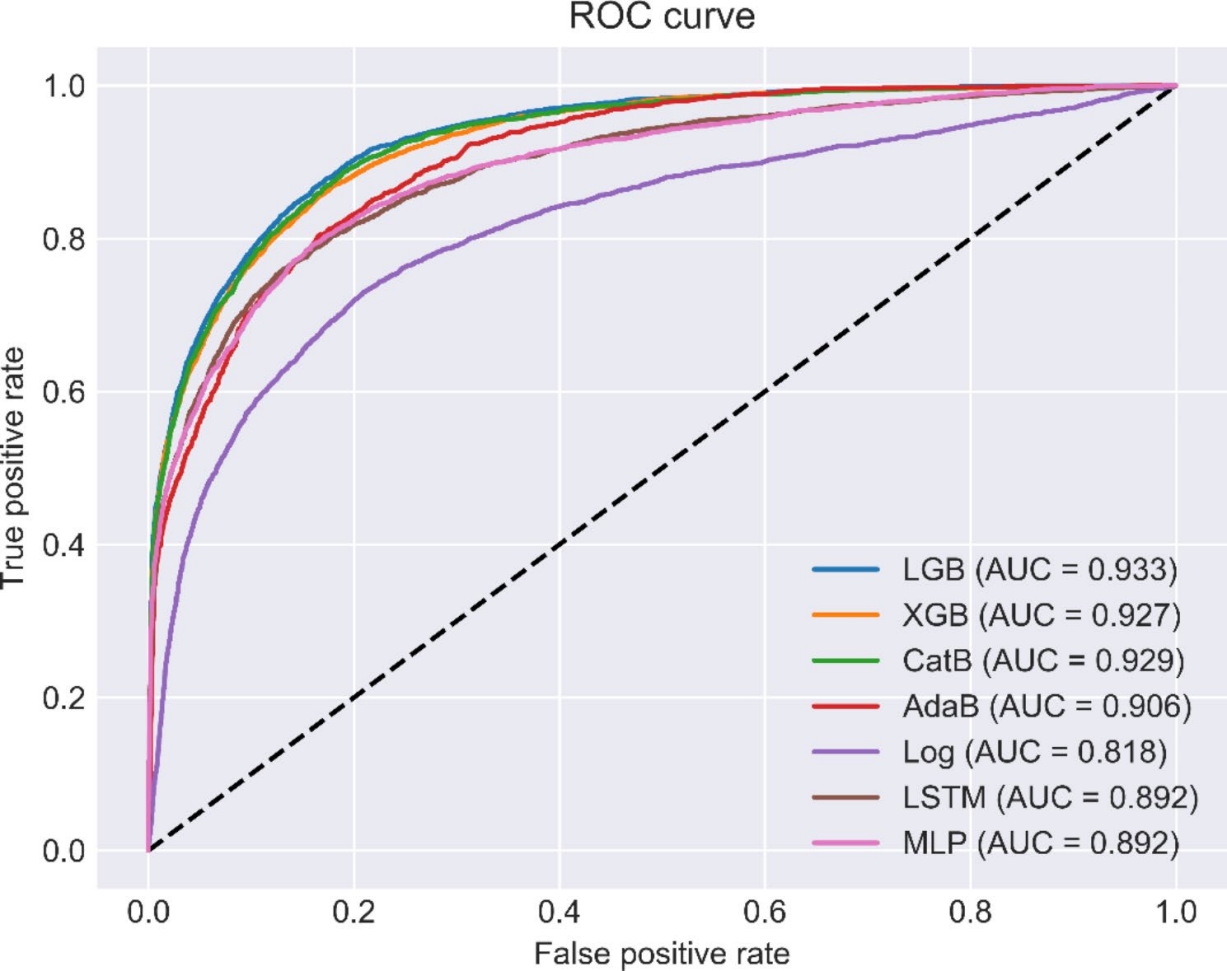
The performance characteristic curves for LGB, XGB, CatB, AdaB, Log, LSTM, and MLP

The discriminative performance of machine learning models is shown in Fig. 3 as expressed by the receiver operating characteristic curves (ROCs) in the training and test datasets. LGB achieved the highest AUC of all the methods, with a value of 0.933 (sensitivity = 0.87, specificity = 0.85, accuracy = 0.87). XGB also achieved the second-highest AUC, with a value of 0.927 (sensitivity = 0.85, specificity = 0.85, accuracy = 0.85). The performance of the models for the pooled dataset is described in Table 2. LGB outperformed all seven comparison methods, as measured by AUC, sensitivity, specificity and accuracy.

**Table 2:** Performance of machine learning models of AUC, sensitivity, specificity, and accuracy

Model	AUC	Sensitivity	Specificity	Accuracy
**LGB**	**0.933**	**0.87**	**0.85**	**0.87**
XGB	0.927	0.85	0.85	0.85
CatB	0.929	0.86	0.83	0.84
AdaB	0.906	0.67	0.84	0.77
LR	0.818	0.41	0.83	0.65
MLP	0.892	0.79	0.84	0.83
LSTM	0.892	0.8	0.83	0.73

**Fig. 4:**
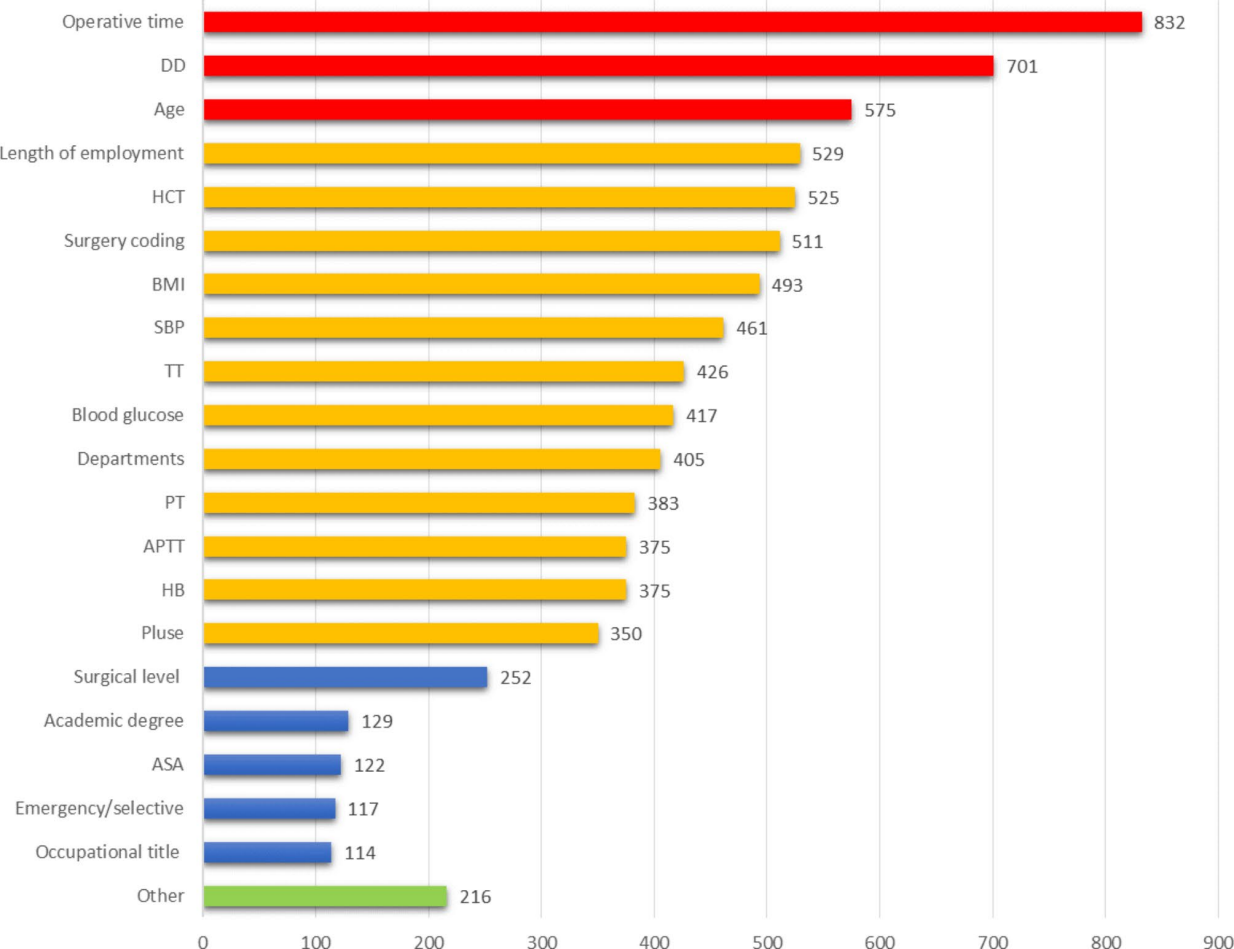
Standard feature importance bar chart shows the importance of each predictor in the LGB model

Standard feature importance bar charts (Fig. 4) give a notion of relative importance. Operative time, DD, and age before surgery were the first three features to predict the end of bleeding. Other significant predictors included length of employment, hematocrit, surgery coding, BMI, SBP, TT, blood glucose, departments, PT, APTT, hemoglobin, and pulse.

**Fig. 5:**
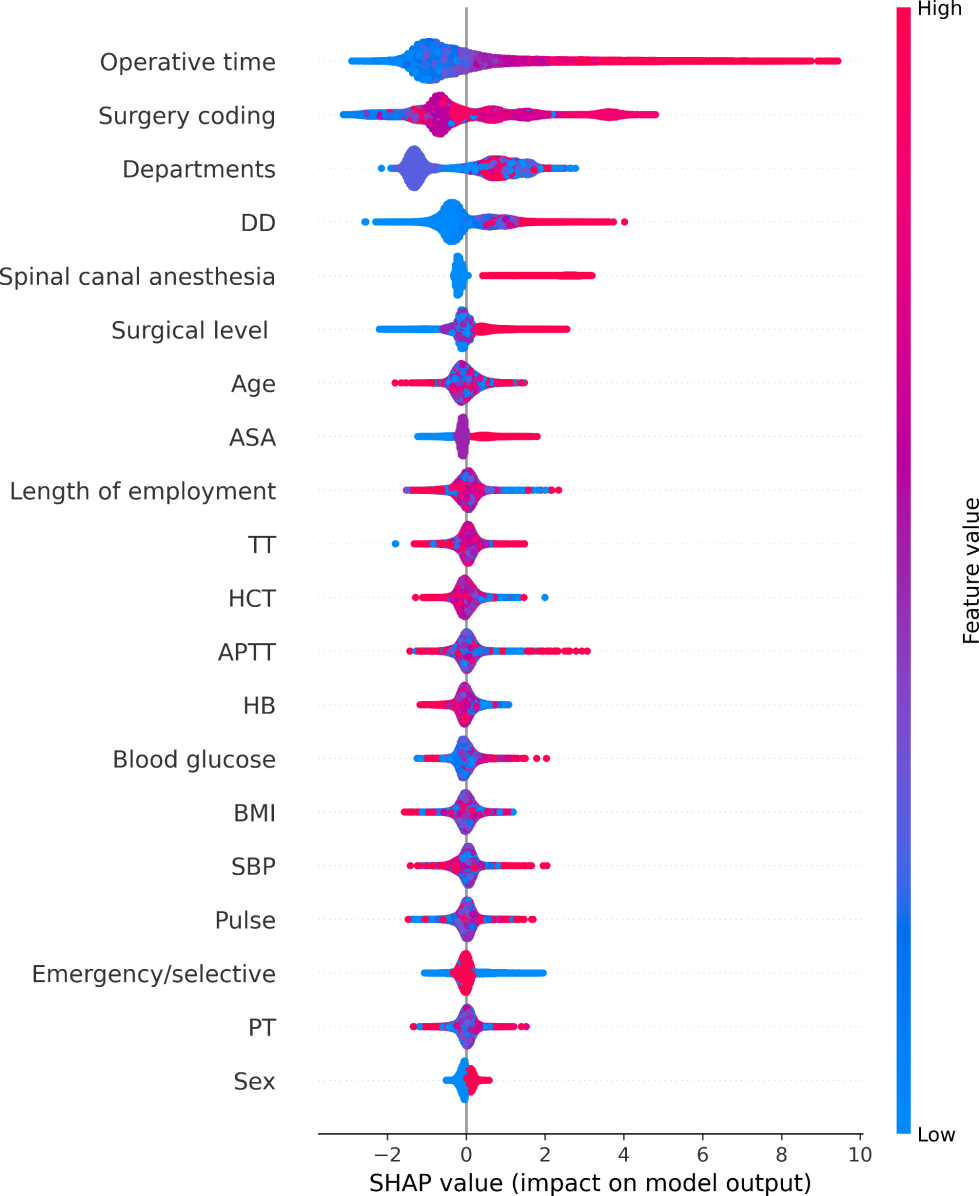
SHAP summary plots of a 20-feature LGB prediction model on intraoperative bleeding RWD

SHAP summary plots (Fig. 5) leverage individualized feature attributions to express the range and distribution of a feature [30]. We can directly see the impact of each characteristic on the prediction of bleeding risk. Red to blue represents the eigenvalue from large to small. The thickness of the line represents the sample distribution. The higher the SHAP value of a feature, the higher your log odds of risk. At the same time, in the analysis of various characteristics, the impact of each factor on risk can be analyzed one by one. As shown in Fig. 5, bleeding risk was roughly proportional to operative time, and operative time was the most important risk factor for intraoperative bleeding. The density of the operative time plot shows how common different operative times are in the dataset. It has a large impact on a minority of people with long operative times, and the risk of bleeding increases by approximately 80 min. The general trend of long tails reaching the right means that extreme values of operative time can significantly raise the risk of bleeding. In contrast to operative time, DD has a ‘pure’ impact on a majority of people. The trend of tails reaching the left means that low values can significantly lower your risk. According to age, there was no obvious trend in most middle-aged people, but there was an increased risk in younger patients.

## Discussion

In this study, we used RWD on 48,543 inpatients for surgery to generate and test machine learning models to predict the risk for intraoperative bleeding. Our baseline statistics showed that 20.04% of patients experienced bleeding. The LGB model presented excellent discriminative abilities. Operative time, DD, and age were the top few important variables, which could guide further preoperative preparation and surgical optimization and identify high-risk patients by a personalized evaluation.

We explored 27 variables, most of which were routinely assessed from preoperative examination and EMRs during the management of patients admitted for surgeries. The easy access to data facilitated clinical application.

We found that operative time acted as the most valuable predictor in the LGB model. Operative time has been confirmed as a significant and independent risk factor associated with intraoperative bleeding [32]^,^ [33]. The risk of massive intraoperative bleeding was significantly higher in long surgery patients than in short surgery patients [33]. A linear relationship was observed between operative time and transfusions (indicating excessive blood loss) when the operative time exceeded 75 to 80 min [34]. Anirudh K Gowd also considered that a 15-minute increase in operative duration was associated with an increased risk of transfusion [35]. In our study, surgery coding may contribute to the operative time and intraoperative bleeding. Therefore, the operative time should be shortened as much as possible, and the preoperative international normalized ratio (INR) value should be controlled [36] to reduce the risk of bleeding [32].

Length of employment ranks fourth in the LGB model, and it is an interesting variable worth discussing. Our results showed that surgeons with longer employment years presented lower intraoperative bleeding risk than those with shorter employment years. Some studies considered that the surgeons’ experience cannot decrease bleeding for the following reason [4, 37]: with experience, the surgeon performs more complex cases that involve more risk of intraoperative bleeding [2]. However, it may reflect under-adjustment for risk, unmeasured confounding by traditional statistics. LGB may avoid interaction and potential confounding among variables, and demonstrate better agreement with human intuition.

Older and younger patients in this study were more likely to manifest as intraoperative bleeding than middle-aged patients. Advanced age may be associated with hypertension or malignant diagnoses, an increased likelihood of prescription medication use, or weak tolerance of the procedure, which cause a high rate of intraoperative bleeding [38]. However, the risks of intraoperative bleeding in younger patients were not adequately considered in previous studies. Jeon et al. found that younger age was a significant predictor of intraoperative bleeding during endoscopic operations, but the causes were still not discussed [39]. In our study, the higher intraoperative bleeding risk may be related to the surgery type. Younger patients were more likely to experience surgeries of the musculoskeletal system, which are usually accompanied by mass bleeding. Sex was not of great importance in the standard feature importance bar charts, but it is shown in the SHAP. Compared with males (left of the boundary), females faced a higher risk of bleeding, and the color of SHAP was ‘pure’ red. A study confirmed an increased risk of in-hospital bleeding in women [40]. Physiological mechanisms of coagulation could change with a greater tendency of bleeding due to menstruation and pregnancy [41], but the female factor is considered to have little influence in our model.

There were some important biomarkers, DD and hematocrit, for the prediction of inoperative blood loss. DD was positively correlated with total blood loss on a postoperative day by a generalized linear model [42], which is similar to our GBDT models. Our results mainly focus on a low concentration of DD and low bleeding risk. It is convenient to apply low-dose oral Xa inhibitors and is thought to have a lower risk of bleeding [43]. However, there is evidence of an association between the risk of thromboembolic disease and DD > 0.5 µg/mL [44]. When adjusting DD preoperatively, physicians should step on the balance beam between the risk of embolism and bleeding. Elevating the hematocrit could shorten the bleeding time [45]. In classification and regression tree analyses, hematocrit ≥ 44% was associated with larger estimated blood loss [46]. Preoperative hematocrit counts may identify patients at increased bleeding risk.

Some researchers hold the view that LR is one of the best-performing machine learning models in developing risk-scoring systems [47]. However, LR has greatly reduced performance in dealing with nonlinear problems. It is difficult to address the situation of data imbalance and fit the distribution of RWD. LR is sensitive to outliers, and this can be a disadvantage when dealing with data that has a large number of outliers. Lastly, LR may face variable selection and over-fitting problems when dealing with large sample size data.

A variety of machine learning algorithms, including LR, boosting, and neural networks, have been widely applied in crowd models in terms of treatment and prognosis [48, 49]. LGB [50] is a type of gradient boosting [25] and has been introduced into medical fields in recent years [51]^,^ [52]. From the results of this study, LGB showed the best performance in the intraoperative bleeding outcome in this paper. LGB, XGB, and CatB are almost unanimous in terms of AUC, but LGB has a better recall rate, which can screen out more patients with high intraoperative bleeding risk. There is little research about LGB and intraoperative bleeding, but the application in other disease fields demonstrates the good performance of LGB, such as Parkinson’s disease diagnosis [53] and the prediction of diabetes mellitus [54]. Efficiency is a key advantage of LGB, which is optimized for both training speed and memory usage. This makes LGB particularly suitable for predicting perioperative bleeding, especially which often involves large datasets and complex features. Automatic feature transformation is a powerful feature of LGB, which is particularly useful when predicting perioperative bleeding, as it often involves multiple features that may be of different types. LGB uses histogram-based algorithms to transform them into more informative representations. By doing so, LGB can effectively enhance the prediction accuracy, making it a valuable tool. Robustness to missing values is a key advantage of LGB in medical applications, where data often contain missing values. LGB can handle missing values automatically without requiring additional data preprocessing steps, making it a valuable tool in predicting perioperative bleeding. λFeature importance metrics is a valuable feature of LGB in predicting perioperative bleeding. By providing insights into the contribution of each feature to the model’s predictions, LGB’s feature importance metrics enable better understanding and interpretation of the model.

The recall rate of bleeding patients detected by LGB has reached 90%, which greatly improved the early warning rate of high-risk patients. The value of XGB in postpartum hemorrhage prediction reached 0.93 [55], and the AUC was 0.91 in upper gastrointestinal bleeding [56], which is similar to our study. Therefore, the LGB model is recommended as the optimal model for the prediction of surgical patients with a high risk of intraoperative bleeding.

In recent years, there has been a growing trend in the field of model development, which involves combining traditional methods with machine learning algorithms. One approach involves using regression models to screen features and select those with a high correlation to bleeding risk [13]. The selected features are then ranked using a machine learning algorithm to obtain their importance. This approach aims to enhance the overall performance and interpretability of the model through feature screening and ranking. However, this approach has some limitations. The selection of features may be subjective and human-biased, as it is based on domain knowledge or experience. It may not capture all relevant features or overlook important ones. While the method combines linear regression and machine learning, it may not be an accurate fit for complex data patterns and may not handle non-linear relationships well. To better understand the advantages and disadvantages of these methods, we summarize them in Table 3.

**Table 3:** A comparison of the advantages and disadvantages of the three methods

	Traditional models	HMLS	Machine learning algorithms
Strengths	Simple, easy to understand and master	Improve extrapolation and interpretation of models	1. Applicable to datasets with diverse distribution 2. Robust to outliers and missing values 3. Allow for direct feature selection 4. Avoid over-fitting 5.Strong extrapolation capabilities
Weaknesses	1. The sensitivity to outliers 2. A weak feature selection ability 3. Overfitting 4. Limited ability to extrapolate beyond the available data	1. Filter out irrelevant features depends on prior knowledge and expertise. 2. Weak ability to handle non-linear relationships 3.May not fit accurately for complex data patterns.	Filter out irrelevant features depends on prior knowledge and expertise

Our study has several strengths that increase the validity and impact of our findings. Firstly, we had a large sample size and collected comprehensive data, which enabled robust statistical analysis and increased the generalizability of our results. Additionally, we overcame the challenge of class imbalance by applying techniques that have been ignored in other studies. Furthermore, our study focused on a clinically relevant outcome that has important implications for patient care and management. Finally, our study provides practical guidance for clinicians and researchers who are interested in using prediction models to identify patients at high risk of bleeding.

This study has several limitations. First, this is RWD, so some potential variables are not available in the current study. Second, we did not establish a follow-up cohort, and postoperative bleeding could not be monitored. Third, we derived these data only from a single tertiary hospital in Shanghai, so the generalizability of the results is unpredictable. Extended validation would proceed in prospective data and other hospitals. In addition, the results and improvement measures of this study will be applied in our clinical practice to observe the actual results in the real world.

## Conclusion

GBDT algorithms, especially LGB, appear to be efficient tools to assess the risk of intraoperative bleeding in surgical adult patients. Several principal predictors for bleed were linked to surgical procedure and patient characteristics and should receive attention. The prediction model can be used to optimize surgeries and decrease bleeding. Further validation in prospective data is needed for extended application.

### Electronic supplementary material

Below is the link to the electronic supplementary material.

Supplementary Material 1

Supplementary Material 2

## Data Availability

The datasets used and/or analyzed during the current study are available from. the corresponding author on reasonable request.

## Declarations

### Author information

Ying Shi and Guangming Zhang co-first author.

## Abbreviations

HMLS: Hybrid machine learning systems
EMRs: Electronic medical records
AUC: Area under the receiver operating characteristic curve
LGB: Light gradient boosting
XGB: Extreme gradient boosting
CatB: Cathepsin B
AdaB: Ada-boosting of decision tree
LR: Logistic regression
MLP: Multilayer perception
LSTM: Long short-term memory
DD: D-dimer
GBDT: Gradient Boosting Decision Tree
RWD: Real-world data
BMI: Body mass index
SHAP: SHapley Additive exPlanation
SD: Standard deviation
ROCs: Receiver operating characteristic curves
SBP: Systolic blood pressure
TT: Thrombin time
PT: Prothrombin time
APTT: Activated partial thromboplastic time
