# Supplementary material for: Machine learning algorithms to predict intraoperative hemorrhage in surgical patients: a modeling study of real-world data in Shanghai, China
Source: BMC Med Inform Decis Mak. 2023 Aug 10;23:156. doi: 10.1186/s12911-023-02253-w (PMC10416513; doi:10.1186/s12911-023-02253-w)
Supplement: Supplementary file 1 — Supplementary Material 1 [file 12911_2023_2253_MOESM1_ESM.docx]

[Appendix table 1 Input features used for training the machine learning model](#appendix_table1)s

| Epidemiological/clinical features | Sex |
| --- | --- |
|  | Age |
|  | BMI |
| Underlying illnesses | Kidney/heart/hypertension/diabetes/ coagulopathy |
| Surgical features | Surgery coding |
|  | Surgical level (Ⅰ/Ⅱ/Ⅲ/Ⅳ) |
|  | Elective/Emergency procedures |
|  | Anesthesia method |
|  | ASA |
|  | Operative time |
| Information of the surgeon | Occupational title |
|  | Departments |
|  | Length of employment |
|  | Academic degrees |
| Biochemical criterion | Pulse |
|  | Systolic blood pressure (SBP) |
|  | Blood glucose |
|  | D-dimers (DD) |
|  | Hemoglobin (HB) |
|  | Hematocrit (HCT) |
|  | Thrombin time (TT) |
|  | Prothrombin time (PT) |
|  | Activated Partial thromboplastic time (APTT) |
