# Supplementary material for: Machine learning algorithms to predict intraoperative hemorrhage in surgical patients: a modeling study of real-world data in Shanghai, China
Source: BMC Med Inform Decis Mak. 2023 Aug 10;23:156. doi: 10.1186/s12911-023-02253-w (PMC10416513; doi:10.1186/s12911-023-02253-w)
Supplement: Supplementary file 2 — Supplementary Material 2 [file 12911_2023_2253_MOESM2_ESM.docx]

[Appendix table 2 Categorization of anesthesia modalities](#appendix_table2)

| Model variable | Variable in HIS |
| --- | --- |
| Spinal canal anesthesia | Subarachnoid block anesthesia  (lumbar anesthesia; spinal anesthesia) |
|  | Epidural block anesthesia |
|  | CSEA; combined spinal-epidural anesthesia |
| General anesthesia | Inhalation anesthesia |
|  | total intravenous anesthesia |
|  | Bronchial general anesthesia |
| Nerve block | Sciatic nerve block |
|  | Cryptorhystem block |
|  | Femoral nerve block |
|  | Adductor block |
|  | Iliac fascial nerve block |
|  | Closed-for-hole nerve block |
|  | Popliteal nerve block |
|  | Lateral femoral cutaneous nerve block |
|  | Cervical plexus anesthesia |
|  | Brachial plexus anesthesia |
|  | Transverse abdominal muscle plane block technology (TAP) |
|  | Transverse abdominal fascia block (TEP) |
|  | Erector Spinal Plane Block (ESP) |
|  | Intercostal nerve block |
|  | Rectus abdominis sheath block (Rectus abdominis block) |
|  | Anterior serrative nerve block |
|  | Abdominal nerve block |
|  | Supra laryngeal nerve block |
| Local anesthesia | Surface anesthesia |
|  | Other |
| Monitored Anesthesia (MAC) | MAC (conscious sedation) |
|  | conscious sedation |
|  | Basal anesthesia |
